# Moderately prolonged permissive hypotension results in reversible metabolic perturbation evaluated by intracerebral microdialysis - an experimental animal study

**DOI:** 10.1186/s40635-019-0282-x

**Published:** 2019-12-04

**Authors:** Rasmus Peter Jakobsen, Troels Halfeld Nielsen, Simon Mølstrøm, Carl-Henrik Nordström, Asger Granfeldt, Palle Toft

**Affiliations:** 1 Department of Anesthesia and Intensive Care, Odense University Hospital, J.B. Winsløws Vej 4, Indgang 8, 20, 201, 5000 Odense C, Denmark; 2 Department of Neurosurgery, Odense University Hospital, Kløvervænget 47, Indgang 44, 1. etage, 5000 Odense C, Denmark; 3 Department of Intensive Care, Aarhus University Hospital, Palle Juul-Jensens Blvd. 99 G304, 8200 Aarhus, Denmark

**Keywords:** Hemorrhage, Shock, Microdialysis, Cerebrum, Permissive hypotension, Lactate, Pyruvate, Venous microdialysis

## Abstract

**Background:**

Damage control resuscitation (DCR) and damage control surgery (DCS) is the main strategy in patients with uncontrollable hemorrhagic shock. One aspect of DCR is permissive hypotension. However, the duration of hypotension that can be tolerated without affecting the brain is unknown. In the present study we investigate the effect of 60 min severe hypotension on the brain’s energy metabolism and seek to verify earlier findings that venous cerebral blood can be used as a marker of global cerebral energy state.

**Material and methods:**

Ten pigs were anaesthetized, and vital parameters recorded. Microdialysis catheters were placed in the left parietal lobe, femoral artery, and superior sagittal sinus for analysis of lactate, pyruvate, glucose, glycerol, and glutamate. Hemorrhagic shock was induced by bleeding the animal until mean arterial pressure (MAP) of 40 mmHg was achieved. After 60 min the pigs were resuscitated with autologous blood and observed for 3 h.

**Results:**

At baseline the lactate to pyruvate ratios (LP ratio) in the hemisphere, artery, and sagittal sinus were (median (interquartile range)) 13 (8–16), 21 (18–24), and 9 (6–22), respectively. After induction of hemorrhagic shock, the LP ratio from the left hemisphere in 9 pigs increased to levels indicating a reversible perturbation of cerebral energy metabolism 19 (12–30). The same pattern was seen in LP measurements from the femoral artery 28 (20–35) and sagittal sinus 22 (19–26). At the end of the experiment hemisphere, artery and sinus LP ratios were 16 (10–23), 17 (15–25), and 17 (10–27), respectively. Although hemisphere and sinus LP ratios decreased, they did not reach baseline levels (*p* < 0.05). In one pig hemisphere LP ratio increased to a level indicating irreversible metabolic perturbation (LP ratio > 200).

**Conclusion:**

During 60 min of severe hypotension intracerebral microdialysis shows signs of perturbations of cerebral energy metabolism, and these changes trend towards baseline values after resuscitation. Sagittal sinus microdialysis values followed hemisphere values but were not distinguishable from systemic arterial values. Venous (jugular bulb) microdialysis might have a place in monitoring conditions where global cerebral ischemia is a risk.

## Background

Trauma remains a leading cause of mortality. The leading cause of preventable deaths in trauma patients is uncontrollable hemorrhage [1]. During recent years, damage control resuscitation (DCR) and damage control surgery (DCS) has been the main strategy in the management of major hemorrhage in trauma patients [2, 3]. Damage control resuscitation incorporates balanced blood transfusions, tranexamic acid, and permissive hypotension until surgical hemostasis or control is achieved [4–7]. The brain is the most sensitive organ in regard to hypotension, and knowledge, of the effect of permissive hypotension on the brain without traumatic brain injury is scarce [8–10]. The authors have in a previously published experimental study shown that prolonged (90 min) and severe hypotension (mean arterial pressure (MAP) of 40 mmHg) results in irreversible metabolic perturbation evaluated by intracerebral microdialysis (MD) [11]. Furthermore, the same study showed that the cerebral biochemical changes in response to severe hypotension were reflected in the cerebral venous outflow. The primary aim of the present study is to investigate the effect of moderately prolonged (60 min) severe hemorrhagic shock (MAP = 40 mmHg) on the brain’s metabolism evaluated by microdialysis, and secondly to confirm our previously findings that venous microdialysis can be used as a marker of global energy metabolism. The hypothesis is that moderately prolonged and severe shock causes reversible metabolic derangement defined as a temporary increase in hemisphere lactate to pyruvate ratio (LP ratio).

## Materials and methods

The study was approved by the Danish Animal Experiments Inspectorate (2015–15–0201–00788). The depth and duration of hemorrhagic shock necessary for producing cerebral ischemia that caused compromised energy state was based on previous experiments [11] and 4 initial experiments that confirmed feasibility. This was followed by six additional female pigs approximately 4 months old. All were Danish landrace mix, Yorkshire Duroc breed. The median weight was 42 (35–45) kg (interquartile range).

### Anesthesia, mechanical ventilation, and surgical preparation

The porcine model of hemorrhagic shock has been previously described [12]. The animals fasted overnight with access to ad libitum water. Sedation was achieved with a standard combination of medetomidine (0.05 mg/kg), midazolam (0.25 mg/kg), and atropine (0.25 mg/kg). Anesthesia was induced with midazolam (0.625 mg/kg) and ketamine (12.5 mg/kg) and maintained with an infusion of midazolam (5 mg/kg/h) and fentanyl (50 μg/kg/h). The animals were intubated and volume-controlled ventilated (Siemens 900 Ventilator; Siemens Elema, Stockholm, Sweden) with, a tidal volume of 10 mL/kg and FiO2 of 0.30. PaCO2 was kept between 4 and 6 kPa and body temperature around normal 38.5 °C. Crystalloid intravenous fluid 2–4 mL/kg/h was administered until start of the experimental protocol.

### Multimodal monitoring

After establishing anesthesia one sheath was inserted into the carotid artery for blood pressure monitoring and blood gas sampling. The external jugular vein was cannulated for the insertion of a central venous catheter to deliver anesthesia. Arterial blood gases (PaCO2, PaO2, pH), blood glucose, electrolytes, and lactate levels were measured every 30 min. (epoc vet, Alere, Waltham, MA, USA).

One femoral artery was cannulated for withdrawing and re-infusing blood during the induced hemorrhagic shock. Another sheath was placed in the contralateral femoral artery, and a microdialysis catheter (CMA 67, MDialysis, Stockholm, Sweden) was inserted through a standard 18G IV catheter. A small craniotomy was placed in the frontal bone in the midline above the superior sagittal sinus. The sinus was cannulated by an 18G peripheral venous catheter, and one microdialysis catheter (CMA 70 Bolt MDialysis, Stockholm, Sweden) was introduced in a posterior direction and placed in the posterior part of the superior sagittal sinus. The superior sagittal sinus was chosen for analysis of cerebral venous blood due to the anatomic characteristics of the experimental animal. In the pig, most of the cerebral blood is drained via paraspinal venous plexa and only a minor part passes into the internal jugular vein [13]. A third microdialysis catheter (CMA 70, MDialysis AB, Stockholm, Sweden) was inserted 20 mm into the left parietal lobe, and one probe for monitoring brain tissue oxygenation (PbtO_2_) (Licox CC1SB, Integra Neurosciences Ltd. New Jersey, USA) was introduced 15 mm into the contralateral parietal lobe. A transducer for monitoring intracranial pressure (ICP) (Camino, Integra Neurosciences Ltd. New Jersey, USA) was placed in the right hemisphere. After insertion, all probes were allowed a minimum of 1 h for stabilization. A bladder catheter was placed for urine collection. All animals were given a baseline dose of 200 U/kg of heparin and supplemented hourly with 100 U/kg for anticoagulation during the hemorrhage period. At the end of the experiment, the anesthetized animals were killed with an intravenous injection of sodium pentobarbital 200 mg/mL in concentrated ethanol.

### Experimental protocol

Following a 60-min baseline period allowing animals to stabilize after surgery hemorrhagic shock was achieved by bleeding the animals to a pre-defined MAP of approximately 40 mm Hg at a rate of 2.15 mL/kg/min over 7 min, and then 1.15 mL/kg/min over the remaining period [12]. Animals were kept at a MAP of about 40 mmHg by withdrawing or infusing shed blood that was stored in a citrated glucose solution at 37 °C. Following 60 min of hemorrhagic shock, the animals were resuscitated by re-infusing the shed blood at a rate of 120 mL/min until all blood was returned. The pigs were observed for 3 h after hemorrhagic shock. Microdialysis probes were perfused with artificial CSF (M Dialysis AB, Stockholm, Sweden) at a rate of 0.3 μL/min (CMA 106 MD pump, MDialysis AB, Stockholm, Sweden). The dialysates were collected in microvials and immediately analyzed for glucose, lactate, pyruvate, glutamate, and glycerol every 30 min using an Iscus Flex analyzer (M Dialysis AB, Stockholm, Sweden).

PbtO_2_ data were collected using the AC3.1 monitor (Integra Neurosciences Ltd.) and recorded every 20 s. All Licox probes were tested against atmospheric air and against each other before insertion and after removal. After insertion, the appropriate function was confirmed by an oxygen challenge test.

ICP was monitored continuously and data were collected by a CAM01 monitor (Integra Neurosciences Ltd. New Jersey, USA) and ICP. Cerebral perfusion pressure (CPP) was calculated as MAP-ICP.

## Statistics

**Fig. 1:**
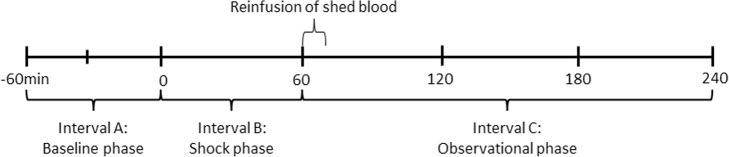
Experimental protocol timeline. Note that the experimental protocol is divided into three intervals. Interval A: baseline phase; interval B: shock phase with a mean arterial pressure of 40 mmHg; and interval C: observational phase after resuscitation with shed blood

Data are given as median (interquartile range) unless otherwise noted. To test our hypothesis, the time course of the experimental protocol was divided into three intervals: Fig. 1 interval A which consists of the baseline prior to hypotension; interval B, during hypotension; and interval C, after resuscitation with shed blood. The end of hypotension was defined post hoc as the time when PbtO_2_ had increased to baseline values. To test the hypothesis data were modeled utilizing a mixed effect model for repeated measurements with time interval as fixed effect and each animal as random effect. Each animal functioned as its own control. The null hypothesis was that no difference in the hemisphere LP ratio was found between interval A and C. To test our hypothesis regarding global sagittal sinus microdialysis, the time course of the LP ratio in the superior sagittal sinus and femoral artery was also modeled utilizing a mixed effect model with time and location of microdialysis probe as random effects and each animal as fixed effect. The null-hypothesis was that no difference in the LP ratio was found between the superior sagittal sinus and femoral artery. *p* values below 0.05 was considered significant. Data analysis was performed in Stata 15 statistical software (StataCorp, College Station, Texas, USA).

## Results

All 10 animals completed the protocol. However, one animal showed signs of irreversible metabolic perturbation during the experiment indicated by very high levels of LP ratio (> 200) and low levels of glucose and brain tissue oxygenation. This animal was analyzed separately.

The sagittal sinus MD catheter of three of the animals showed low levels (< 20 mmol/L) of glutamate indicating that the catheters had dislodged into the subdural space. The data from these MD catheters were excluded.

General physiological and biological variables are listed in Table 1.

**Table 1:** General physiological and biochemical variables doing hemorrhagic shock. *N* = 9. Data are expressed as median levels (interquartile range) during the experimental protocol intervals A, B, and C. *MAP* mean arterial pressure, *ICP* intracranial pressure, *CPP* cerebral perfusion pressure, *PbtO*_*2*_ brain tissue oxygenation. S indicates start of hemorrhage. Time 0 indicates achievement of MAP equal 40 mmHg. Test statistics made with mixed effect model

Elapsed time (min)	Baseline phase Interval A (− 60)–0 min	Shock and resuscitation phase Interval B 0–70 min	Observational phase Interval C 70–220 min	Interval A versus interval C *p* value
MAP (mmHg)	95 (66–107)	39 (35–42)	76 (67–84)	*p* < 0.005*
ICP (mmHg)	8 (6.5–11)	6 (5–9)	10 (7–14)	*p* = 0.089
CPP (mmHg)	84 (60–97.5)	33 (28–39)	65 (53–79.5)	*p* < 0.005 *
PbtO2 (kPa)	29 (24–32)	19 (8–23)	35 (20–45)	*p* < 0.005*
PaO2 (kPa)	24 (22–26)	24 (23–25)	22 (22–24)	*p* = 0.002*
PaCO2 (kPa)	5.4 (5,5–89)	5.1 (4,7–5.6)	6.1 (5.7–6.7)	*p* = 0.025*
b-hemoglobin (mM/L)	8.6 (5.8–9.8)	7.9 (5.2–8.8)	7.8 (6–9.3)	*p* = 0.084
HR (bpm)	80 (71–92)	125 (82–152)	81 (72–92)	*p* = 0.502
b-Glucose (mM/L)	5.5 (4.3–7.2)	6.3 (4.2–9.8)	5.3 (4.1–6.9)	*p* = 0.010*
b-lactate (mM/L)	0.9 (0.5–1.2)	4.3 (2.5–7.3)	1.8 (0.9–5.1)	*p* = 0.009*
b-pH	7.47 (7.45–7.51)	7.44 (7.39–7.44)	7.40 (7.32–7.46)	*p* = 0.249
Diuresis (mL)	55 (10–160)	3 (0–12)	19 (6–32)	*p* < 0.005*

*Statistical significant *p* value < 0.05

The median volume of shed blood was 1469 mL (1378.4–1583.44 mL) and median blood loss per kilogram was 35 mL/kg (31.9–36.8 mL/kg).

**Fig. 2:**
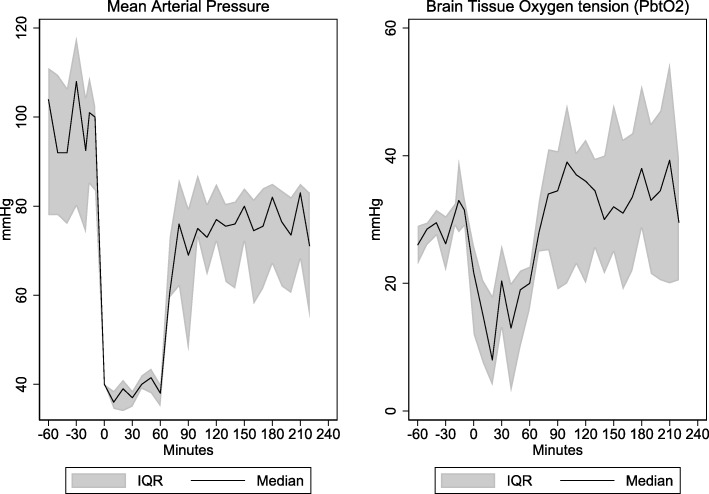
Mean arterial pressure and brain tissue oxygen tension. Median (interquartile range) arterial pressure (MAP) and brain tissue oxygen tension (PtbO2) in pigs (*n* = 9) with induced hemorrhagic shock. Note that during the period of hemorrhagic shock the declining MAP was accompanied by a decrease in PbtO_2_ near critical levels (< 15 mmHg). After re-infusion of autologous blood MAP increased to close to baseline level whereas PbtO_2_ increased to levels above baseline. Time 0 indicates achievement of MAP equal to 40 mmHg

During the period of hemorrhagic shock, MAP decreased to a median value of 39 mmHg (35–42). Accordingly, CPP decreased to low levels of 33 mmHg (28–39). Similarly, the brain tissue oxygen tension decreased to near critical levels of 19 mmHg (8–23). After resuscitation with shed blood MAP increased to near baseline levels (*p* < 0.05). The CPP showed a similar response, normalizing, but not reaching baseline levels (*p* < 0.05). In contrast, PbtO_2_ increased to levels exceeding baseline values and stayed at that level during the rest of the observation period (*p* < 0.05). The relationship between MAP and PbtO_2_ can be seen in Fig. 2. Blood gases, pH, and blood glucose were all kept within normal range.

### Microdialysis values

Measurements obtained by microdialysis are listed in Table 2.

**Table 2:** Biochemical variables obtained from microdialysis. *N* = 9. Data expressed as (interquartile range) during the experimental protocol intervals A, B, and C. *LP* lactate/pyruvate ratio. S indicates start of hemorrhage. Time 0 indicates achievement of MAP equal 40 mmHg. Test statistics made with mixed effect model

Elapsed time (min)	Location	Baseline phase Interval A (− 60)–0 min	Shock and resuscitation phase Interval B 0–70 min	Observational phase Interval C 70–220 min	Interval A versus interval C *p* value
LP ratio	Hemisph.	13 (8–16)	19 (12–30)	16 (10–23)	0.020*
	Sag. sinus	9 (6–22)	22 (19–26)	17 (10–27)	0.012*
	Femoral	21 (18–24)	28 (20–35)	17 (15–25)	0.467
Lactate (mM/L)	Hemisph.	2.3 (1.3–3.0)	3.2 (2.5–6.1)	3 (2.3–4.5)	0.000*
	Sag. sinus	0.9 (0.3–2.5)	4.7 (3.4–6.3)	2.6 (1.1–5.4)	0.002*
	Femoral	1.1 (0.8–1.8)	4.6 (1.6–6.1)	1.7 (0.8–3.9)	0.009*
Pyruvate (mM/L)	Hemisph.	142 (98–203)	147 (120–261)	156 (127–249)	0.000*
	Sag. sinus	109 (48–151)	197 (187–313)	153 (113–201)	0.003*
	Femoral	55 (44–75)	151 (45–198)	94 (53–154)	0.000*
Glucose (mM/L)	Hemisph.	2.2 (1.8–3)	1.8 (1.4–3.1)	2.5 (2–3.5)	0.031*
	Sag. sinus	1.9 (1–3)	3.5 (2.4–5.1)	3.5 (2.3–4.5)	0.000*
	Femoral	4.9 (3.4–5.6)	6 (4.9–7.3)	5.5 (4.6–7.1)	0.010*
Glutamate (μM/L)	Hemisph.	15 (4–22)	12 (5–14)	8 (3–11)	0.050
	Sag. sinus	121 (60–178)	115 (94–176)	177 (110–195)	0.011*
	Femoral	195 (172–205)	190 (184–196)	183 (173–231)	0.181
Glycerol (μM/L)	Hemisph.	38 (25–88)	59 (35–116)	77 (58–125)	0.000*
	Sag. sinus	21 (7–55)	69 (43–130)	61 (34–123)	0.006*
	Femoral	15 (12–28)	47 (22–174)	46 (30–209)	0.000*

*Statistical significant *p* value < 0.05

**Fig. 3:**
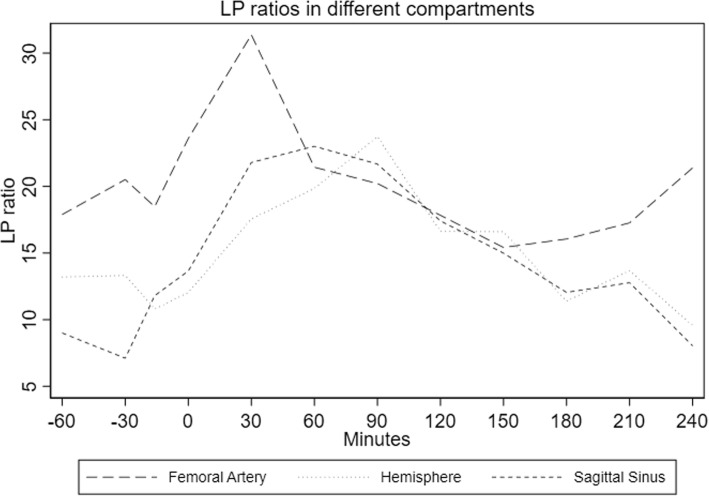
LP ratios in different compartments. LP ratio (median (interquartile range)) in brain parenchyma, venous (superior sagittal sinus), and arterial (femoral artery) blood doing hemorrhagic shock in pigs (*n* = 9). Note that changes in hemisphere LP ratio are paralleled by changes in both venous (sagittal sinus) and arterial blood. There was no difference in LP ratio between arterial and venous blood (*p* = 0.353). Time 0 indicates achievement of MAP equal to 40 mmHg

The dynamic changes in LP ratio in hemisphere, sagittal sinus, and femoral artery are shown in Fig. 3. Before induction of hemorrhagic shock, the LP ratios were similar in the hemisphere, sagittal sinus, and femoral artery. During hemorrhagic shock the LP ratio in the hemisphere increased to levels below the upper normal level for anesthetized piglets ((21 ± 9) (mean ± SD)) [14; 15]. After resuscitation, the LP ratio decreased towards baseline levels but remained elevated throughout the observation period (*p* = 0.02). The same pattern was seen for the sagittal sinus whereas the femoral artery LP ratio returned to baseline values (*p* = 0.467). LP ratio in the femoral artery reached a higher absolute level to a median of 28 (20–35) than the hemisphere or sagittal sinus. However, baseline levels were also higher in the femoral artery. There was no difference in the LP ratio during the experimental protocol between the femoral artery and sagittal sinus (*p* = 0.353).

**Fig. 4:**
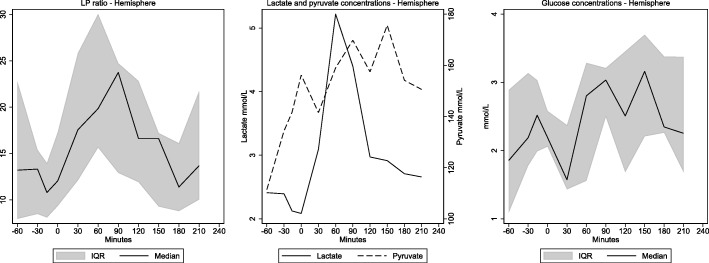
Reversible metabolic perturbation. 60 min hypoperfusion. LP ratio and microdialysis levels of lactate, pyruvate, and glucose (median (interquartile range)) in brain parenchyma doing hemorrhagic shock in pigs (*n* = 9). Note that LP-ratio increases doing hemorrhagic shock but tends to normalize after re-infusion of blood. The rise of LP ratio is due to an increase in lactate levels whereas pyruvate shows a less pronounced elevation. Time 0 indicates achievement of MAP equal to 40 mmHg

The dynamic changes of lactate, pyruvate, and glucose along with changes in LP ratio in the hemisphere are shown in Fig. 4. Lactate baseline values were near the upper normal levels ((1.8 ± 0.8) mean ± SD) [14]. During hemorrhagic shock lactate levels increased to a median value of 3.2 mmol/L (2.5–6.1 mmol/L). After resuscitation hemisphere, lactate levels fell, but not reaching baseline values in the observational period (*p* < 0.05). Pyruvate demonstrated increasing tendency during shock and throughout the observation period. Accordingly, the changes in LP ratio were mainly due to change in lactate.

Hemisphere glucose showed a small increase throughout the experimental protocol (*p* = 0.031) but remained within normal levels ((1.8 ± 0.8) mean ± SD) [14]. The same trend was seen in arterial and sagittal sinus glucose *p* = 0.01 and *p* < 0.05 respectively (Table 2).

Intracerebral glycerol increased slightly during the hypotensive and observational periods to 59 (35–116) μmol/L and 77 (58–125) μmol/L respectively (Table 2). In contrast, hemisphere glutamate fell but remained within normal values throughout the observational period (Table 2).

**Fig. 5:**
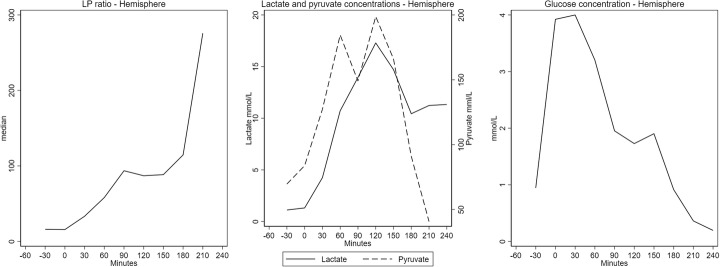
Irreversible metabolic perturbation. 60 min hypoperfusion. LP ratio and microdialysis levels of lactate, pyruvate, and glucose in brain parenchyma doing hemorrhagic shock in pig showing signs irreversible metabolic perturbations. Note that LP ratio rises almost exponentially. This increase is due to a constant elevated lactate level and a temporary rise in pyruvate level. After re-infusion of blood, a marked decrease in hemisphere glucose is seen. Time 0 indicates achievement of MAP equal to 40 mmHg

One animal showed signs of irreversible perturbation of cerebral energy metabolism evaluated by hemisphere microdialysis (Fig. 5). After induction of hemorrhagic shock, the LP ratio increased to very high levels (> 200). The increase was due to a pronounced and lasting elevation of lactate (> 15 mmol/L) and less pronounced but lasting decrease in pyruvate. Along with the increase in LP ratio, a dramatic decrease in glucose (< 1 mmol/L) was seen.

## Discussion

The effect of permissive hypotension on cerebral energy metabolism is not well described. The cerebral energy state is completely dependent on oxidative metabolism, which is reflected immediately in the cerebral cytoplasmic redox state. The ratio between interstitial lactate and pyruvate (LP ratio) is shown to be a robust marker of the cellular redox state.

In the present study we demonstrate that a period of 60 min severe hypotension causes a decrease in PbtO_2_ but only a small increase in the intracerebral LP ratio. This increase along with stable values of glucose, glycerol, and glutamate indicates reversible metabolic perturbation of the cerebral redox state.

### Brain tissue oxygenation (PbtO_2_) and LP ratio

During the induced hemorrhagic shock PbtO_2_ decreased to levels regarded as near critical [15]. Along with the decrease in PbtO_2_ the LP ratio increased but remained within normal levels. The elevation was due to an increase in lactate along with a less pronounced increase in pyruvate.

Although the metabolic perturbation is caused by a decrease blood and oxygen supply, the observed pattern is not consistent with cerebral ischemia. Cerebral ischemia is characterized by a significant decrease in PbtO_2_ and glucose along with a significant increase in LP ratio due to an increase in lactate and decrease in pyruvate [16]. On the other hand, the observed metabolic pattern in the present study is compatible with mitochondrial dysfunction [17]. The concept of mitochondrial dysfunction should be viewed in its broadest term. Mitochondrial dysfunction will obviously exist when mitochondria are damaged, but the metabolic patterns are also seen if the metabolic demands exceed the capacity of the oxidative metabolism, i.e., during seizures [18]. However, yet another mechanism is responsible for the observed metabolic pattern in the present study.

During hemorrhage, numerous compensatory mechanisms are activated on both the cellular, tissue, and whole-organism level. The end goal is to maintain a steady supply of oxygen to end-organ tissues as well as prevent excessive bleeding and ensure hemostasis. As bleeding continues, cardiac output, and eventually blood pressure, drops [19]. To ensure cerebral perfusion pressure and cerebral blood flow (CBF) is adequate, autoregulatory mechanism exists. This autoregulatory mechanism ensures that CPP is regulated within a narrow range. The mechanism has been described as both myogenic, metabolic, and neurogenic [20, 21]. When the blood pressure reaches a point where the delivery of oxygen to peripheral tissue is compromised, transition to anaerobic metabolism occurs. During this gradual decrease in CBF oxygen supply to the brain would be insufficient before the supply of substrate (glucose) is seriously jeopardized. This is because of the difference in concentrations and degree of tissue extraction [22]. This phase between compensated low systemic blood pressure and pronounced cerebral ischemia was characterized as hypoxic hypoxia by Siesjö in the 1970s [22], and is characterized by decreasing PbtO_2_ and elevation of lactate along with normal values of glucose and pyruvate similar to the observed pattern in the present study. As blood pressure continues to decrease deliverance of both oxygen and glucose is further compromised resulting in a pattern of ischemia and metabolic crisis. We have previously shown in a similar animal model that hemorrhagic shock to a MAP of 40 mmHg and a duration of 90 min caused ischemia and subsequent irreversible cerebral damage evaluated by intracerebral microdialysis, ICP, and PbtO_2_ [11]. In the present study, 60 min of hypotension resulted in an apparently reversible metabolic perturbation. Accordingly, the metabolic perturbations during hemorrhagic shock represent a continuum from normal cerebral metabolism to jeopardized but potential reversible metabolism similar to hypoxic hypoxia towards irreversible ischemia.

The pattern of pronounced cerebral ischemia was seen in one animal in the present study and was characterized by a marked increase in intracerebral LP ratio and decrease in PbtO_2_ and glucose. The rise in LP ratio was caused by a steep increase in lactate along with a decrease in pyruvate. This finding underlines the individual differences in each animal regarding cerebral autoregulation and ability to compensate physiological crisis, and that 60 min of severe hypotension is on the edge of how long the autoregulatory mechanisms can compensate for low CBF/systemic blood pressure.

After reinfusion of shed blood, the PbtO_2_ level increased to levels above baseline values. As PbtO_2_ primarily is linked to the CBF [23] this increase is likely due a post-hypoperfusion hyperemia.

Parallel to the increase in PbtO_2_ levels during the observational period, a decrease was seen in LP ratio tending towards baseline levels but not reaching baseline values. Thus, our null hypothesis cannot be rejected. Although the LP ratio did not return to baseline values, the increase in hemisphere LP ration did not exceed normal levels at any point during the experimental protocol, thus supporting reversible metabolic perturbation. This conclusion is supported by the interstitial levels of glutamate and glycerol. Glutamate is regarded as a marker of pending energy failure [24], and glycerol is regarded as a marker of cell membrane degradation and hence cell damage [25]. In the present study glutamate remained stable and within normal values [26]. Glycerol exhibited a slight increase during the observation period but remained within normal values [26].

The microdialysis findings of the present study support the notion that not only the depth but also the duration of the hypotensive period determines if the alterations in brain redox state are reversible or irreversible.

Our findings are supported by an animal study by Wan et al. who reported that intracerebral microcirculation was unaffected by deep and long hemorrhagic hypotension [27]. These findings are also supported in two studies investigating the effect of prolonged and severe hemorrhagic hypotension in rats. The authors found that MAP 40 mmHg for 60–75 min did not cause cognitive damage to the rat, nor was it possible to detect apoptotic areas in the hippocampal area of the brain [28, 29]. However, the abovementioned studies only examined the microcirculatory flow and not the presence of oxygen and nutrients in the interstitial space. Although the authors reported that microcirculation was unaffected, and no structural brain damage was found, deliverance and uptake of oxygen and nutrients might be affected.

### Global microdialysis

Our secondary objective was to verify our earlier findings that venous (superior sagittal sinus) microdialysis can be used as a measure for the hemisphere redox state. During the experimental protocol an increase in hemisphere LP was paralleled by an increase in venous (sagittal sinus) LP ratio, but this rise is not distinguishable from the systemic LP rise. Thus, our secondary null hypothesis cannot be rejected. We have previously demonstrated that global changes in cerebral lactate and pyruvate are reflected in the cerebral venous outflow and distinguished from systemic (i.e., arterial blood) perturbations [11, 30]. However, in the present study a similar increase in lactate and pyruvate was observed in arterial blood. Less pronounced shock leads to less pronounced global metabolic crisis and thus less pronounced alterations in venous LP ratio. During hemorrhagic shock an increase in anaerobic metabolism is present on a whole-organism level. The systemic arterial values of lactate and pyruvate would increase, and thus, an increase in systemic LP ratio would be seen. This systemic increase in LP ratio can mask an increase in global cerebral LP ratio measured in venous blood. After e.g. cardiac arrest where the systemic anaerobe metabolism has normalized to aerobic metabolism, the brain may still be suffering after a metabolic ischemic crisis. During supportive venous-arterial extracorporeal membrane oxygenation, venous microdialysis may also be able to detect unfavorable distribution of watershed line to the cerebrum which is not detected by bifrontal near-infrared spectroscopy (NIRS) [30, 31].

### Clinical implications

The present study demonstrates an increase in hemisphere LP ratio during moderately long and severe shock. By comparing present findings with earlier finding in a study with 90-min duration of shock, this indicates that there is an upper limit for how long the cerebral autoregulatory mechanisms can compensate for a low systemic blood pressure. As demonstrated by one animal which experienced severe cerebral metabolic changes, not only time but also individual differences between each animal affect how long the autoregulatory mechanisms are able to maintain an adequate cerebral perfusion pressure. During damage control resuscitation and permissive hypotension, all is being done to ensure control of hemorrhage and secure the patient’s survival. Although this is being done as fast as possible it is wise to bear in mind that there might be an upper limit in which autoregulatory mechanisms exist. The rise in hemisphere LP ratio during hemorrhagic shock was followed by a rise in the sagittal sinus LP ratio, but this rise is not distinguishable from the systemic LP rise. In a clinical context, the less invasive technique with jugular bulb microdialysis might have a place in advanced monitoring where global cerebral ischemia or isolated cerebral pathologies might be present. When viewing the results from the present and earlier study [11] together this indicates that reversible metabolic crises might not be detected, but as soon as metabolic crises reach a level where the irreversible injury is imminent, it would be detected.

### Limitations

In the present study, we use a fixed-pressure experimental model of hemorrhagic shock. The advantages of this model are its reproducibility as well as the ability of maintaining of a desired target MAP. It also mimics the clinical context where ongoing blood loss is replaced by transfusion with whole blood until a certain target MAP. One disadvantage of the model is the use of heparin to maintain the patency of intravascular catheters before, during, and after hemorrhage. Some studies have shown a possible effect on microcirculation after hemorrhagic shock. However, these studies use substantial higher doses, than in the present study [32]. The use of anesthetics also depresses the animal’s cardiovascular ability to compensate for a decreasing blood pressure. The anesthetized animals also mimic patients undergoing DCS, while utilizing the concepts of DCR. Many of the data collected showed a visual trend towards normalizing, and one might wonder if the values had returned to baseline values if the observation period had been longer. Obviously, the definition of each of the three intervals might affect the statistical test. The definition of each interval, when it starts, and when it ends could also influence the results. Intuitively a rise in LP ratio after a reversible metabolic crisis needs time to return to baseline values after the hypotensive period has ended. In our study we have defined the end of interval B when PbtO_2_ has normalized indicating a non-critical deliverance of oxygen and nutrients to the brain. Although this does not account for the time the brain’s redox status to return to normal, we consider it to be safer to overestimate the effect of injury.

## Conclusion

This experimental study demonstrates the severe (40 mmHg) and moderately prolonged (60 min) hemorrhagic shock results in a reversible metabolic crisis evaluated by microdialysis. The study also demonstrates that while minor perturbations in the cerebral metabolism might be paralleled in the venous outflow through the sagittal sinus, they are indistinguishable from systemic values during systemic ischemia.

Since time apparently is an important factor in the prevention of irreversible brain damage future studies should focus on methods (e.g., vasoactive drugs) to prolong the period in which permissive hypotension is safe for the brain.

## Data Availability

The datasets used and analyzed during the current study are available from the corresponding author on reasonable request.
